# Systematic design methodology for robust genetic transistors based on I/O specifications via promoter-RBS libraries

**DOI:** 10.1186/1752-0509-7-109

**Published:** 2013-10-27

**Authors:** Yi-Ying Lee, Chih-Yuan Hsu, Ling-Jiun Lin, Chih-Chun Chang, Hsiao-Chun Cheng, Tsung-Hsien Yeh, Rei-Hsing Hu, Che Lin, Zhen Xie, Bor-Sen Chen

**Affiliations:** 1 Lab of Control and Systems Biology, Department of Electrical Engineering, National Tsing Hua University, Hsinchu 30013, Taiwan; 2 Lab of Genetic Circuit Design, Department of Electrical Engineering, National Tsing Hua University, Hsinchu 30013, Taiwan; 3 Department of Electrical Engineering, National Tsing Hua University, Hsinchu 30013, Taiwan; 4 Center for Synthetic and Systems Biology, Bioinformatics Division, TNLIST, Tsinghua University, Beijing 100084, China

**Keywords:** Genetic transistor, Input/Output (I/O) characteristics, Promoter-RBS library, Systematic design methodology, Design specifications

## Abstract

**Background:**

Synthetic genetic transistors are vital for signal amplification and switching in genetic circuits. However, it is still problematic to efficiently select the adequate promoters, Ribosome Binding Sides (RBSs) and inducer concentrations to construct a genetic transistor with the desired linear amplification or switching in the Input/Output (I/O) characteristics for practical applications.

**Results:**

Three kinds of promoter-RBS libraries, *i.e.*, a constitutive promoter-RBS library, a repressor-regulated promoter-RBS library and an activator-regulated promoter-RBS library, are constructed for systematic genetic circuit design using the identified kinetic strengths of their promoter-RBS components.

According to the dynamic model of genetic transistors, a design methodology for genetic transistors via a Genetic Algorithm (GA)-based searching algorithm is developed to search for a set of promoter-RBS components and adequate concentrations of inducers to achieve the prescribed I/O characteristics of a genetic transistor. Furthermore, according to design specifications for different types of genetic transistors, a look-up table is built for genetic transistor design, from which we could easily select an adequate set of promoter-RBS components and adequate concentrations of external inducers for a specific genetic transistor.

**Conclusion:**

This systematic design method will reduce the time spent using trial-and-error methods in the experimental procedure for a genetic transistor with a desired I/O characteristic. We demonstrate the applicability of our design methodology to genetic transistors that have desirable linear amplification or switching by employing promoter-RBS library searching.

## Background

Synthetic biology aims to perform various specific functions in organisms by inserting a designed gene network. In the past, synthetic biology could be classified as having two broad purposes. The first was to create artificial life from natural biology using the synthetic methods. The other was to assemble some functional components using interchangeable natural components which are nonexistent in natural biology [1]. A lot of the recent literature focuses on performing electronic circuit behaviors in organisms using genetic devices such as toggle switches [2-5], oscillators [6-13], pulse generators [14,15], logic gates [16-19], and filters [20-22]. Synthetic biologists also design various types of genetic circuits with different functionalities by employing genetic devices to solve useful tasks, such as biosensor decisions or edge detection [23,24].

In many industries, such as the electronics and manufacturing industries, the characterization and standardization of components and the institution of specifications are already key elements in the production line. In the synthetic biology, the expression of a specific protein needs a promoter, a ribosome binding side (RBS), a protein coding sequence and a terminator, which are DNA fragments. The Registry of Biological Standard Parts (http://www.partsregistry.org), formed by MIT, shows many standard BioBricks, and these standard biological components provide synthetic biologists with a quick and standardized way of constructing gene circuits. Also, BioFAB provides some sorts of biobricks (see http://biofab.org/data), enabling the rapid design and prototyping of genetic constructs. However, in the past, the strength of a promoter and an RBS, which are the main components of transcription and translation, were defined according to their relative strength with other promoters and RBSs. Now, however, the promoter-RBS strength can be quantified by measuring the fluorescence of proteins whose coding gene is constructed at the downstream of the promoter-RBS component.

Based on the kinetic strengths of promoter-RBS components, promoter-RBS libraries are constructed for gene circuit design. In our gene circuit design, promoter-RBS libraries are built based on kinetic parameters of the dynamic gene regulation, which are identified by the nonlinear least squares method based on experimental data. We formulate the design specifications of desired gene circuits in advance and choose an adequate set of promoter-RBS components from the promoter-RBS libraries based on the characterized, standardized and quantified components. For our promoter-RBS libraries, we select three kinds of promoters, *i.e.*, constitutive promoters, repressor-regulated promoters and activator-regulated promoters, to combine with RBSs as the promoter-RBS components. Each of these is constructed using the green fluorescent protein in *Escherichia coli*. and characterized to allow for the construction of the following three types of promoter-RBS libraries: constitutive promoter-RBS libraries, repressor-regulated promoter-RBS libraries and activator-regulated promoter-RBS libraries.

To date, several researchers have demonstrated that synthetic gene circuits have the functionality of amplification or switching [25-29]. These gene circuits can amplify the input signal or switch the output signal as it exceeds a specific threshold level. Often shown in these genetic amplifiers is the use of a two-stage cascade of promoters to achieve the function of amplification. However, these circuits only amplify a low level of input signal or low concentration of inducer at the first stage while the second stage consists of the promoter-RBS activity being fixed. On the other hand, switching circuits switch the output signal using an external inducer. When the inducer is externally increased, the circuit is on, and vice versa. Nevertheless, at present, the on-state, or high level, of switching has not been clearly defined.

In this paper, we demonstrate that a simple repressive gene circuit can work like an electrical transistor as an amplifier or a switch. The amplification gains or switch levels of the genetic transistor are regulated by the concentrations of inducer and different combinations of promoters with RBSs. For the convenience of measurement and application, reporter genes are constructed as the measurable input and output. We show that the I/O characteristics of the repressive gene circuit regulated by inducer concentration can be effectively predicted by adequate selection of promoter-RBS components from our libraries. Thus, based on these promoter-RBS libraries, a look-up table is built to quickly select adequate promoter-RBS components for the design of genetic transistors with different design specifications.

In the following sections, we first construct the promoter-RBS libraries based on the promoter-RBS strength through the dynamic regulatory model of promoter-RBS components. Then, we describe the I/O characteristics of a genetic transistor with different kinetic strengths of promoter-RBS components in the promoter-RBS libraries and different concentrations of inducers. Finally, a look-up table (or genetic transistor library) is constructed for genetic transistor design requiring prescribed I/O characteristics, which is used by searching the most appropriate sets of promoter-RBS components and concentrations of inducers via the genetic algorithm (GA).

## Methods

### Construction of the promoter-RBS libraries for genetic transistors

In this section, we introduce the characterization and standardization of promoter-RBS libraries and employ a dynamic mathematical model to construct the promoter-RBS libraries according to the identified kinetic strengths of promoter-RBS components, populated via experimental data.

#### Promoter-RBS libraries based on the identified kinetic strengths of promoter-RBS components

In a systematic design procedure, the characterization and standardization of components are important preparatory tasks before practical design process. These can save designers a significant amount of time and avoid unnecessary trial-and-error attempts. In the field of synthetic biology, a particular technique was developed to create standard interchangeable biological components called BioBricks [30,31]. These allow the synthetic biologists to focus on the design of more complex genetic circuits rather than the basic construction of the gene components.

BioBricks are DNA fragments with specific functions, and include promoters, ribosome binding sites (RBS), repressors, activators, reporters and terminators. In the database, there are only a few BioBrick components that are well-characterized. The well-characterized BioBrick components are conducive to the systematic design of synthetic genetic circuits. In order to facilitate the design of synthetic gene circuits, wider libraries of well-characterized BioBricks need to be constructed.

In our promoter-RBS libraries, the library indexes are the kinetic strengths of promoter and RBS, which are considered together as a promoter-RBS component because the gene expression is regulated by a promoter-RBS component. The kinetic strength of a promoter-RBS component can be systematically identified by a stochastic model which simulates the dynamic behavior of promoter-RBS components under some external molecular or environmental noises. In order to identify the kinetic strength of a promoter-RBS component, the green fluorescence gene is embedded into the downstream of the promoter-RBS component. By measuring the fluorescence dynamic time profile and using the nonlinear least squares method [32], we identify the kinetic strengths of the promoter-RBS components to be used as the indexes of promoter-RBS libraries.

The construction procedure of the promoter-RBS libraries can be generally divided into four steps [33]: (i) choose the required promoter-RBS components, (ii) select the suitable reporter protein and growth conditions, (iii) measure the time-profile data of the dynamic behavior, and (iv) construct the dynamic regulatory model for identifying kinetic strengths of promoter-RBS components to be used as library indexes according to the nonlinear least squares method. In the first step, some promoters can be regulated by specific transcription factors, and different combinations of promoters with RBSs give different kinetic strengths of promoter-RBS components, which increase the diversity of the libraries. In order to rapidly obtain a variety of kinetic strengths of promoter-RBS components, a mutation technique was used to create different kinetic strengths of promoters and RBSs to increase the varieties of promoter-RBS components through the mutation of a specific region on promoters or RBSs [18,33]. In the second and third step, since different reporter proteins, such as the green fluorescent protein or red fluorescent protein have different degradation rates, the measurement times may differ. Further, the cell growth conditions have an effect on the results of the measurement. Biological component can be characterized at different cellular growth phases, under different culture conditions, or at different resolutions. In our experiment, the GFP is selected as the reporter protein and the time profiles of fluorescence are measured by the microplate reader. In the final step, a mathematical dynamic model is built to describe the time profile of protein expression. Using the protein expression time profile measurements, the nonlinear least squares method is employed to identify the kinetic strengths of promoter-RBS components to be used as the library indexes with the mathematical model. For the systematic design of genetic transistors, we construct three kinds of promoter-RBS libraries, *i.e.*, constitutive, repressor-regulated and activator-regulated promoter-RBS libraries. The promoter-RBS components in promoter-RBS libraries and all BioBrick components used in this study are listed in Additional file 1, respectively. The detailed construction procedures of constitutive, repressor-regulated and activator-regulated promoter-RBS libraries are described in Additional file 1.

### Construction and design of the genetic transistor

After the introduction of regulatory functions of promoter-RBS components and the construction of the promoter-RBS libraries, we design a synthetic genetic circuit, similar to a transistor, with prescribed I/O characteristics of amplification or switching through the external inducer. Before the construction of the synthetic genetic transistor, we introduce the simply operation of an electronic transistor in Additional file 1 to which the genetic transistor will be designed accordingly following.

#### Construction of the genetic transistor

**Figure 1:**
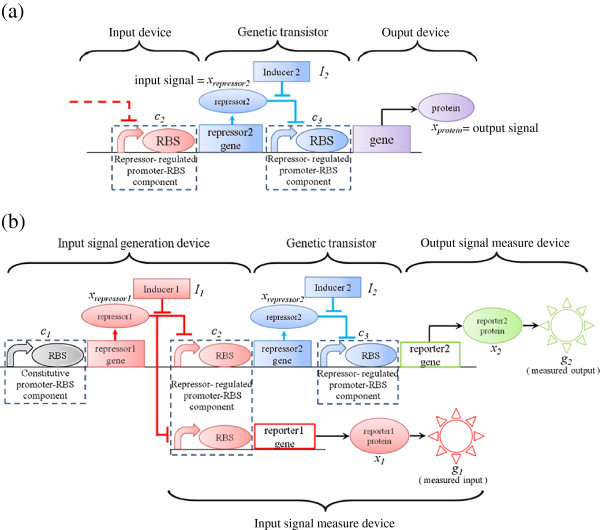
**The representation of synthetic genetic transistor circuit. (a)** A genetic transistor. **(b)** A genetic transistor with measurement circuit. The input signal of the genetic transistor is measured by RFP reporter and the output signal of the genetic transistor is measured by GFP reporter.

A genetic transistor is shown in Figure 1(a). The transistor is constructed to obtain the output protein concentration *x*_*protein*_ of the transistor for amplification or switching behavior. The genetic transistor consists of the repressor-regulated promoter-RBS component *c*_3_ and a repressor coding gene. The input repressor *x*_*repressor*2_ to the genetic transistor is controlled by the repressor-regulated promoter-RBS component *c*_2_, which is regulated by the corresponding repressor. The input repressor *x*_*repressor*2_ will form the complex and restrict the production of output protein *x*_*protein*_ by binding the corresponding repressor-regulated promoter-RBS component *c*_3_ to decrease its kinetic strength. However, when the inducer is added, this inducer will bind input repressor *x*_*repressor*2_ and prevent it from binding to the repressor-regulated promoter-RBS component *c*_3_. Then, both the kinetic strength of repressor-regulated promoter-RBS component *c*_3_ and the production of output protein *x*_*protein*_ will increase. The dynamic model of a genetic transistor is described as follows:

(1) $$\begin{array}{ll} {\dot{x}}_{\mathrm{repressor}2}\left(c_{2},t\right)= & \;p_{\mathrm{repressor}}\left(P_{M,c_{2}},P_{m,c_{2}},0,0\right)\\ & \;-\left(\mu +\gamma_{\mathrm{repressor}2}\right)x_{\mathrm{repressor}2}\left(c_{2},t\right)\\ {\dot{x}}_{\mathrm{protein}}\left(c_{3},t\right)= & \;p_{\mathrm{repressor}}\left(P_{M,c_{3}},P_{m,c_{3}},x_{\mathrm{repressor}2},I_{2}\right)\\ & \;-\left(\mu +\gamma_{\mathrm{protein}}\right)x_{\mathrm{protein}}\left(c_{3},t\right) \end{array}$$

where *x*_*repressor*2_ and *x*_*protein*_ denote the concentrations of input repressor2 and the output protein of the genetic transistor, respectively, and *γ*_*protein*_ denotes the degradation rate of the protein.

However, the protein concentration is difficult to directly measure and quantify. To determine characteristics of the synthetic genetic transistor, a genetic transistor with measurement circuit is constructed as shown in Figure 1(b). In Figure 1(b), we construct an additional repressor-regulated promoter-RBS component *c*_2_ so that the input reporter protein *x*_1_ can be measured by input fluorescence *g*_1_ and the output reporter protein *x*_2_ can be measured by output fluorescence *g*_2_. Note that RFP is used to measure input while GFP is used to measure output. Additionally, for the convenience of the input regulation, we construct an input signal generation device with the concentration of inducer *I*_1_ to control the input *g*_1_ of the genetic transistor circuit. Then, the dynamic model of a synthetic genetic transistor circuit with I/O measure devices under environmental disturbances is described by the following set of equations:

(2) $$\begin{array}{l} \left\{\begin{array}{l} \begin{array}{l} {\dot{x}}_{\mathrm{repressor}1}\left(c_{1},t\right)=p_{\mathrm{const}}\left(P_{c_{1}}\right)-\left(\mu +\gamma_{\mathrm{repressor}1}\right)x_{\mathrm{repressor}1}\left(c_{1},t\right)\\ \;+v_{1}\left(t\right) \end{array}\\ \begin{array}{l} {\dot{x}}_{\mathrm{repressor}2}\left(c_{2},t\right)=p_{\mathrm{repressor}}\left(P_{M,c_{2}},P_{m,c_{2}},x_{\mathrm{repressor}1},I_{1}\right)\\ \;-\left(\mu +\gamma_{\mathrm{repressor}2}\right)x_{\mathrm{repressor}2}\left(c_{2},t\right)+v_{2}\left(t\right) \end{array}\\ \begin{array}{l} {\dot{x}}_{1}\left(c_{2},t\right)=p_{\mathrm{repressor}}\left(P_{M,c_{2}},P_{m,c_{2}},x_{\mathrm{repressor}1},I_{1}\right)\\ \;-\left(m_{1}+\mu +\gamma_{\mathrm{im},x_{1}}\right)x_{1}\left(c_{2},t\right)+v_{3}\left(t\right) \end{array}\\ {\dot{g}}_{1}\left(c_{2},t\right)=m_{1}\cdot x_{1}\left(c_{2},t\right)-\left(\mu +\gamma_{m,x_{1}}\right)g_{1}(c_{2},t)+v_{4}(t)\\ \begin{array}{l} {\dot{x}}_{2}\left(c_{3},t\right)=p_{\mathrm{repressor}}\left(P_{M,c_{3}},P_{m,c_{3}},x_{\mathrm{repressor}2},I_{2}\right)\\ \;-\left(m_{2}+\mu +\gamma_{\mathrm{im},x_{2}}\right)x_{2}\left(c_{3},t\right)+v_{5}\left(t\right) \end{array}\\ {\dot{g}}_{2}\left(c_{3},t\right)=m_{2}\cdot x_{2}\left(c_{3},t\right)-\left(\mu +\gamma_{m,x_{2}}\right)g_{2}\left(c_{3},t\right)+v_{6}(t), \end{array}\right)\\ \;c_{1}\in \mathrm{Li}b_{\mathrm{const}},\;c_{2}\;\mathrm{and}\;c_{3}\in \mathrm{Li}b_{\mathrm{repressor}} \end{array}$$

where *m*_1_ and *m*_2_ denote the maturation rates of reporter1 *x*_1_ and reporter2 *x*_2_, respectively, and *v*_i_(*t*), *i* = 1, 2,   ⋯, 6 denote the noises.

To explore the I/O characteristics of a synthetic genetic transistor with the function of amplification or switching, the steady state model of (2) is given by

(3) $$\begin{array}{l} \left\{\begin{array}{l} x_{\mathrm{repressor}1}\left(c_{1}\right)=p_{\mathrm{const}}\left(P_{c_{1}}\right)/\left(\mu +\gamma_{\mathrm{repressor}1}\right)+v_{s_{1}}\\ \begin{array}{l} x_{\mathrm{repressor}2}\left(c_{2},I_{1}\right)=p_{\mathrm{repressor}}\left(P_{M,c_{2}},P_{m,c_{2}},x_{\mathrm{repressor}1},I_{1}\right)/\\ \;\left(\mu +\gamma_{\mathrm{repressor}2}\right)+v_{s_{2}} \end{array}\\ \begin{array}{l} x_{1}\left(c_{2},I_{1}\right)=p_{\mathrm{repressor}}\left(P_{M,c_{2}},P_{m,c_{2}},x_{\mathrm{repressor}1},I_{1}\right)/\\ \;\left(m_{1}+\mu +\gamma_{\mathrm{im},x_{1}}\right)+v_{s_{3}} \end{array}\\ g_{1}\left(c_{2},I_{1}\right)=m_{1}\cdot x_{1}\left(c_{2}\right)/\left(\mu +\gamma_{m,x_{1}}\right)+v_{s_{4}}\\ \begin{array}{l} x_{2}\left(c_{3},I_{1},I_{2}\right)=p_{\mathrm{repressor}}\left(P_{M,c_{3}},P_{m,c_{3}},x_{\mathrm{repressor}2},I_{2}\right)/\\ \;\left(m_{2}+\mu +\gamma_{\mathrm{im},x_{2}}\right)+v_{s_{5}} \end{array}\\ g_{2}\left(c_{3},I_{1},I_{2}\right)=m_{2}\cdot x_{2}\left(c_{3}\right)/\left(\mu +\gamma_{m,x_{2}}\right)+v_{s_{6}}, \end{array}\right)\\ \;c_{1}\in \mathrm{Li}b_{\mathrm{const}},\;c_{2}\;\mathrm{and}\;c_{3}\in \mathrm{Li}b_{\mathrm{repressor}} \end{array}$$

where $v_{s_{i}}$, *i* = 1, 2,   ⋯, 6 denote the noises at the steady state.

From (3), if *m*_1_ ≈ *m*_2_, $\gamma_{m,x_{1}}\approx \gamma_{m,x_{2}}$ and $\gamma_{\mathrm{im},x_{1}}\approx \gamma_{\mathrm{im},x_{2}}$, then the I/O characteristic can be regarded as input/output = *x*_*repressor*2_/*x*_*protein*_ ≈ *x*_1_/*x*_2_ ≈ *g*_1_/*g*_2_, i.e., we could use the *x*_1_/*x*_2_ or *g*_1_/*g*_2_ ratio to replace the I/O characteristic of the synthetic genetic transistor. Further, the I/O characteristic can be controlled and regulated by the selection of promoter-RBS components *c*_3_ and inducer concentration *I*_2_. Therefore, we need to define the I/O characteristic of synthetic genetic transistor circuits to design a genetic transistor with the desired I/O characteristic. This is done as follows:

(4) $$y_{\mathrm{ss}}\left(c_{3},I_{2},g_{1}\left(c_{2},I_{1}\right)\right)=g_{2}\left(c_{3},I_{1},I_{2}\right),g_{1}\in \left[g_{1e},\;g_{1n}\right]\;\left(a.u.\right)$$

where *y*_*ss*_(*c*_3_, *I*_2_, *g*_1_) denotes the I/O response of the synthetic genetic transistor circuit between input signal *g*_1_ and output signal *g*_2_, and *g*_1e_ and *g*_1*n*_ denote the lower bound and upper bound of *g*_1_. a.u. stands for arbitrary unit.

In Figure 1(b), promoter-RBS components *c*_1_ and *c*_2_ can be selected to control input signals *x*_*repressor*1_(*c*_1_) and *x*_*repressor*2_(*c*_2_, *I*_1_) in (3). In general, genetic components are inherently uncertain in the biological system as a result of gene expression noises in transcription or translation processes, thermal fluctuations, DNA mutations, evolutions, context-dependence between promoters, 5′UTRs, and coding sequences, as well as parameter estimation errors [34-37]. Hence, we model the uncertain kinetic strengths of promoter-RBS components, degradation rate of proteins and transcription/translation rates as stochastic processes in the following model:

(5) $$\begin{array}{l} P_{c_{1}}\to P_{c_{1}}+\Delta P_{c_{1}}n_{1}\left(t\right),P_{M,c_{2}}\to P_{M,c_{2}}+\Delta P_{M,c_{2}}n_{2}\left(t\right),P_{m,c_{2}}\to P_{m,c_{2}}+\Delta P_{m,c_{2}}n_{2}\left(t\right),\\ P_{M,c_{3}}\to P_{M,c_{3}}+\Delta P_{M,c_{3}}n_{3}\left(t\right),P_{m,c_{3}}\to P_{m,c_{3}}+\Delta P_{m,c_{3}}n_{3}\left(t\right),\\ \gamma_{\mathrm{repressor}1}\to \gamma_{\mathrm{repressor}1}+\Delta \gamma_{\mathrm{repressor}1}n_{1}\left(t\right),\gamma_{\mathrm{repressor}2}\to \gamma_{\mathrm{repressor}2}+\Delta \gamma_{\mathrm{repressor}2}n_{2}\left(t\right),\\ \gamma_{\mathrm{im},x_{1}}\to \gamma_{\mathrm{im},x_{1}}+\Delta \gamma_{\mathrm{im},x_{1}}n_{2}\left(t\right),\;\gamma_{m,x_{1}}\to \gamma_{m,x_{1}}+\Delta \gamma_{im,x_{1}}n_{2}\left(t\right),\\ \gamma_{\mathrm{im},x_{2}}\to \gamma_{\mathrm{im},x_{2}}+\Delta \gamma_{\mathrm{im},x_{2}}n_{3}\left(t\right),\;\gamma_{m,x_{2}}\to \gamma_{m,x_{2}}+\Delta \gamma_{m,x_{2}}n_{3}\left(t\right),m_{1}\to m_{1}+\Delta m_{1}n_{2}\left(t\right),\\ m_{2}\to m_{2}+\Delta m_{2}n_{3}\left(t\right),\mu \to \mu +\Delta \mu n_{1}\left(t\right) \end{array}$$

where $\Delta P_{c_{1}}$, $\Delta P_{M,c_{2}}$, $\Delta P_{m,c_{2}}$, $\Delta P_{M,c_{3}}$, $\Delta P_{m,c_{3}}$, Δ*γ*_*repressor*1_, Δ*γ*_*repressor*2_, $\Delta \gamma_{\mathrm{im},x_{1}}$, $\Delta \gamma_{m,x_{1}}$, $\Delta \gamma_{\mathrm{im},x_{2}}$, $\Delta \gamma_{m,x_{2}}$, Δ*m*_1_, Δ*m*_2_ and ∆*μ* denote the standard deviations of stochastic parameters to be tolerated and could be specified before design and *n*_*i*_(*t*), *i* = 1, 2, 3 denote Gaussian noises with zero mean and unit variance. Therefore, $\Delta P_{c_{1}}$, $\Delta P_{M,c_{2}}$, $\Delta P_{m,c_{2}}$, $\Delta P_{M,c_{3}}$, $\Delta P_{m,c_{3}}$, Δ*γ*_*repressor*1_, Δ*γ*_*repressor*2_, $\Delta \gamma_{\mathrm{im},x_{1}}$, $\Delta \gamma_{m,x_{1}}$, $\Delta \gamma_{\mathrm{im},x_{2}}$, $\Delta \gamma_{m,x_{2}}$, Δ*m*_1_, Δ*m*_2_ and ∆*μ* denote the deterministic parts of parameter variations and *n*_*i*_(*t*), *i* = 1, 2, 3 denote different random fluctuation sources. For robust design of the genetic transistor circuit, these parameter fluctuations in (5) will henceforth be considered in the design procedure so that the synthetic genetic transistor can tolerate these kinds of parameter fluctuations *in vivo*.

With fixed concentration of inducer *I*_2_, we expect that the input signal *g*_1_/output signal *g*_2_ (I/O) characteristics of the synthetic genetic transistor in (4) would be similar to the voltage I/O characteristics of the electronic transistor shown in Additional file 1. When the inducer concentration *I*_1_ increases, the kinetic strength of promoter-RBS component *c*_2_ increases along with the fluorescence of the input signal *g*_1_, which means that the repressor concentration *x*_*repressor*2_ increases. Due to the fixed concentration of inducer *I*_2_, the redundant repressors *x*_*repressor*2_, which are not bound by the inducer *I*_2_, will repress the promoter-RBS component *c*_3_, and the fluorescence of output signal *g*_2_ will decrease. Therefore, the I/O characteristic of the synthetic genetic transistor is similar to Additional file 1. Additionally, from Additional file 1, we see that if input signal is in the operation range of linear amplification, the input signal would be inversely amplified.

Now, consider the alternative viewpoint, *i.e.*, the voltage I/O characteristics of an electronic transistor. When *R*_2_/*R*_1_ increases, the reverse amplification gain will become large and the operation region of linear amplification will narrow as shown in (B1)-(B3) and Additional file 1. In the synthetic genetic transistor, we expect that when the concentration of inducer *I*_2_ changes as per the *R*_2_/*R*_1_ ratio in (B2)-(B3), the I/O characteristics would be similar to the voltage I/O characteristics of electronic transistor in Additional file 1. Due to different concentrations of inducer *I*_2_, the effect of the inducer on the input repressor can vary. When the inducer concentration *I*_2_ decreases, the I/O characteristics would sharpen, so the reverse amplification gain becomes large in the operation region of linear amplification.

Finally, when *R*_2_/*R*_1_ is large enough in (B2)-(B3), the operation region of linear amplification will become too narrow and result in a sharp change in this region. Correspondingly with a synthetic genetic transistor, when the inducer concentration *I*_2_ is low enough, the input signal *g*_1_ will produce a small variation, and the output signal *g*_2_ will have an acute change like a switch. Therefore, according to the analysis above, we could obtain varying reverse amplification gains and switch levels by changing the concentration of inducer *I*_2_.

#### Systematic design of a genetic transistor based on design specification

According to the above analysis in Figure 1(b), we can obtain different reverse amplification gains or switch behaviors via regulation of different concentrations of inducer *I*_2_. Additionally, due to the output signal *g*_2_ being under the controlled by promoter-RBS component *c*_3_, we could change the output range by selecting different repressor-regulated promoter-RBS components *c*_3_ from the repressor-regulated promoter-RBS libraries. In this way, we can control the I/O characteristics of a synthetic genetic transistor to obtain different reverse amplification gains or switch levels by choosing different concentrations of inducer *I*_2_ and selecting different repressor-regulated promoter-RBS components *c*_3_ from the repressor-regulated promoter-RBS libraries.

In Figure 1(b), the input signal generation device consists of a constitutive promoter-RBS component *c*_1_, and a repressor-regulated promoter-RBS component *c*_2_ and an inducer *I*_1_. The constitutive promoter-RBS component *c*_2_ is selected to produce the input repressor continually. Further, for convenience of design, the repressor-regulated promoter-RBS component *c*_2_ is selected from the corresponding promoter-RBS library to have sufficient kinetic strength to obtain an adequate maximum regulation range of input signal regulated by inducer *I*_1_. However, the operation region of linear amplification is still limited in the amplifier design of genetic transistor, and the input signal range might not be fully contained in the operation region of linear amplification. Therefore, the input signal range should be considered in relation to the design purpose. In the procedure of amplifier design, the input operation range *g*_1_ ∈ [*g*_1,*l*_, *g*_1,*u*_]  can be set by transforming the inducer concentration *I*_1_ into the input fluorescence according to (2) or (3) as follows

Input operation range:

(6) $$\;I_{1}\in \left[I_{1,l},I_{1,u}\right]\Rightarrow g_{1}\in \left[g_{1,l},g_{1,u}\right]\;\left(a.u.\right)$$

where *I*_1_ and *g*_1_ denote the inducer concentration and input fluorescence, respectively, *I*_1,*l*_ and *I*_1,*u*_ denote the lower and upper bound of inducer concentrations, respectively, and *g*_1,*l*_ and *g*_1,*u*_ denote the lower and upper bound of input fluorescences respectively.

Note that, in the future, when the promoter-RBS libraries are large enough, the promoter-RBS components *c*_1_ and *c*_2_ can be designed and selected to match the input operation range. However, due to the limited size of our promoter-RBS libraries and for the convenience of design, we will select the repressor-regulated promoter-RBS component *c*_2_ from the corresponding promoter-RBS library.

From the above analysis, the design purpose of an amplifier will lead to the selection of a suitable repressor-regulated promoter-RBS component *c*_3_ from the repressor-regulated promoter-RBS libraries and concentration of inducer *I*_2_, *i.e.*, {*c*_3_, *I*_2_}, so that the I/O characteristics of the synthetic genetic transistor in (4) in a specific input range *g*_1_ ∊ [*g*_1,*l*_, *g*_1,*u*_] can match the desired I/O response similar to (B2), *i.e.*,

(7) $$y_{d}\left(g_{1}\right)=\mathrm{gain}\cdot \left(g_{1}-g_{1,l}\right)+g_{2,u},g_{1}\in \left[g_{1l},\;g_{1u}\right]\;\left(a.u.\right)$$

where *g*_1,*l*_ and *g*_2,*u*_ denote the lower bound of input fluorescence *g*_1_ and upper bound of output fluorescence *g*_2_, respectively, and *gain* denotes the amplification gain of the genetic transistor.

On the other hand, the switching behavior will occur when the input signal has a small variation (see Additional file 1), *i.e.*, a high level signal can be switched into a low level signal and vice versa. In the switching behavior of synthetic genetic transistor, each promoter-RBS component has its own basal level. Thus, when the input signal increases, the output signal will rapidly decrease to the basal level. Therefore, the desired I/O response of a switch is described as follows:

(8) $$y_{d}\left(g_{1}\right)=L_{s}+\frac{\left(H_{s}-L_{s}\right)}{1+{\left(g_{1}/g_{t}\right)}^{2}},g_{1}\in \left[g_{1l},\;g_{1u}\right]\;\left(a.u.\right)$$

where *H*_*s*_ and *L*_*s*_ denote the high level and low level of switching, respectively, and *g*_*t*_ denotes the transition point of input fluorescence. Moreover, the input signal range of I/O characteristics of the switch can be set by (6).

Finally, for matching the desired I/O response of an amplifier or switch, the genetic algorithm (GA) is employed to select an adequate repressor-regulated promoter-RBS component *c*_3_ in the repressor-regulated promoter-RBS libraries and the concentration of inducer *I*_2_ to minimize the following cost function [38], respectively, *i.e.*,

(9) $$\begin{array}{l} \min_{\;c_{3}\in \mathrm{Li}b_{\mathrm{repressor}},I_{2}\in \left[I_{2,l},I_{2,u}\right]}J\left(c_{3},I_{2}\right)\\ =\min_{\;c_{3}\in \mathrm{Li}b_{\mathrm{repressor}},I_{2}\in \left[I_{2,l},I_{2,u}\right]}E\int_{g_{1,l}}^{g_{1,u}}{\left(y_{\mathrm{ss}}\left(c_{3},I_{2},g_{1}\right)-y_{d}\left(g_{1}\right)\right)}^{2}dg_{1} \end{array}$$

To summarize the above design procedure of a biological amplifier and switch, a genetic transistor design procedure of by the promoter-RBS library searching method using GA is proposed as follows [38]:

1. Construct the genetic transistor circuit such as in Figure 1(a).

2. Build the dynamic and steady state mathematical model in (2) and (3), respectively.

3. Provide the design specification of amplifier with the desired I/O response as in (6) and (7) or switch with the desired I/O response as in (6) and (8).

4. Provide the standard deviations of parameter fluctuations and environmental disturbances to be tolerated *in vivo* in (5).

5. Minimize the cost function *J*(*c*_3_, *I*_2_) in (9) by selecting an optimal set {*c*_3_, *I*_2_} via GA.

Based on the design procedure of a genetic transistor using the promoter-RBS library searching method with GA, the promoter-RBS component *c*_3_ is selected from the corresponding repressor-regulated promoter-RBS library and the inducer concentration *I*_2_ is selected within [*I*_2,*l*_, *I*_2,*u*_], while the cost function is calculated in each iteration of the selection process. Then, GA would select the most adequate promoter-RBS component *c*_3_ from the corresponding repressor-regulated promoter-RBS library and inducer concentration *I*_2_ ∊ [*I*_2,*l*_, *I*_2,*u*_] to minimize the cost function.

## Results

### *In silico* synthetic genetic transistor design examples based on promoter-RBS libraries

We have presented the construction and design procedure of a synthetic genetic transistor. In this section, the synthetic genetic transistor is designed and simulated to verify the I/O characteristics of amplification and switching. Subsequently, based on our promoter-RBS libraries, the amplification gain in a specific input operation range and switching level are designed by employing GA to select the most adequate promoter-RBS components and inducer concentrations. Finally, to support future application of this method, a look-up table for genetic transistors is built for different genetic transistor design specifications.

#### Amplifier design example of synthetic genetic transistor

**Figure 2:**
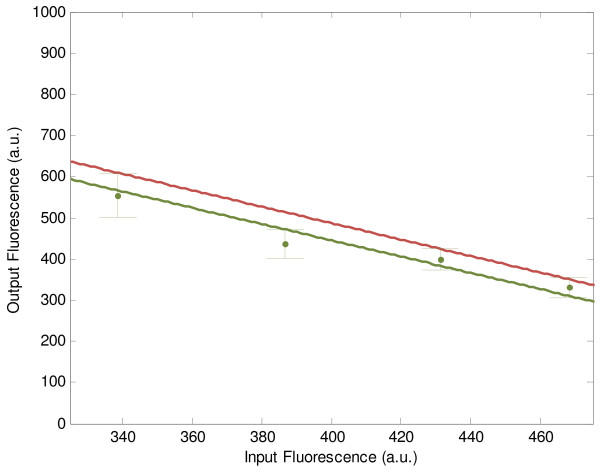
**The amplifier design example of synthetic genetic transistor.** For amplifier design example of synthetic genetic transistor, the prescribed amplification gain = -2 within the range *g*_1_ ∊ [298, 431] is computed by (11) (red line). The most adequate promoter-RBS component *c*_3_ and aTc concentration *I*_*aTc*_ to fit the prescribed amplification gain are searched as {*c*_3_, *I*_*aTc*_} = {*T*_3_, 0 ng/ml} by minimizing *J*_*A*_(*c*_3_, *I*_*aTc*_) in (16) from the corresponding promoter-RBS library *Lib*_*Tet*_ and concentration range of inducer *I*_*aTc*_. The green points are the experimental results based on {*c*_3_, *I*_*aTc*_}, and the error bars are the standard deviations. The green line is the estimation of I/O response of the synthetic genetic transistor based on experimental data, with the estimated amplification gain = -1.978. Obviously, the amplification gain of the designed genetic transistor could match the desired amplification gain quite well.

Consider the amplifier design of the synthetic genetic transistor. Firstly, to obtain the I/O characteristics of amplifier, promoter-RBS components {*c*_1_, *c*_2_} = {*J*_6_, *L*_3_} are selected to obtain the maximum input operation range. The dynamic model and the steady state model have been described in (2) and (3). The input operation range and desired I/O response of genetic transistor are specified as follows:

Input operation range:

(10) $$\;I_{1}\in \left[1.5*10^{-2},4*10^{-2}\right]\;\left(\mathrm{mM}\right)\Rightarrow g_{1}\in \left[298,431\right]\;\left(a.u.\right)$$

and

(11) $$y_{d}\left(g_{1}\right)=-2\cdot g_{1}+1286$$

where -2 is the desired amplification gain as shown in Figure 2. Note that the standard deviations of parameter fluctuations that are supposed to be tolerated *in vivo* are given by

(12) $$\begin{array}{l} \Delta P_{c_{1}}=0.05P_{c_{1}},\left\{\Delta P_{M,c_{i}},\Delta P_{m,c_{i}}\right\}=\left\{0.05P_{M,c_{i}},0.05P_{m,c_{i}}\right\},i=2,3\\ \Delta \gamma_{\mathrm{LacI}}=0.05\gamma_{\mathrm{LacI}},\Delta \gamma_{\mathrm{TetR}}=0.05\gamma_{\mathrm{TetR}}\\ \Delta \gamma_{\mathrm{im},x_{1}}=0.05\gamma_{\mathrm{im},x_{1}},\Delta \gamma_{m,x_{1}}=0.05\gamma_{m,x_{1}}\\ \Delta \gamma_{\mathrm{im},x_{2}}=0.05\gamma_{\mathrm{im},x_{2}},\;\Delta \gamma_{m,x_{2}}=0.05\gamma_{m,x_{2}}\\ \Delta m_{1}=0.05m_{1},\Delta m_{2}=0.05m_{2},\Delta \mu =0.05\Delta \mu \end{array}$$

and the environmental disturbances *v*_*i*_(*t*) are independent Gaussian noises with zero mean and unit variance. Finally, GA is employed to search a set {*c*_3_, *I*_*aTc*_} from corresponding libraries to minimize the following cost function:

(13) $$\begin{array}{l} \min_{\;c_{3}\in \mathrm{Li}b_{\mathrm{Tet}},I_{2}\in \left[1.5*10^{-2},4*10^{-2}\right]}J_{A}\left(c_{3},I_{\mathrm{aTc}}\right)\\ =\min_{\;c_{3}\in \mathrm{Li}b_{\mathrm{Tet}},I_{2}\in \left[1.5*10^{-2},4*10^{-2}\right]}\;E\int_{298}^{431}{\left(y_{\mathrm{ss}}\left(c_{3},I_{\mathrm{aTc}},g_{1}\right)-y_{d}\left(g_{1}\right)\right)}^{2}dg_{1} \end{array}$$

Then, the most adequate promoter-RBS component from the corresponding library and aTc concentration are found to be {*c*_3_, *I*_*aTc*_} = {*T*_3_, 0 ng/ml}. The estimation of I/O response of genetic transistor based on experimental results is shown in Figure 2, with experimental details summarized in Additional file 1. Clearly, the I/O characteristics of genetic transistor can match the desired I/O response in a workable input range *g*_1_ ∊ [298, 431] under the intrinsic fluctuations and environmental disturbances.

#### Switch design example of synthetic genetic transistor

Consider the switch design of the synthetic genetic transistor. The switch design procedure is similar to the amplifier design procedure of a synthetic genetic transistor. Firstly, to obtain the complete I/O characteristics of switching, promoter-RBS components {*c*_1_, *c*_2_} = {*J*_6_, *L*_3_} are selected to obtain the maximum input operation range. The dynamic model and the steady state model have been described in (2) and (3), respectively. The input operation range and desired I/O switch response are specified as follows:

Input operation range:

(14) $$\;I_{1}\in \left[2*10^{-3},10\right]\;\left(\mathrm{mM}\right)\Rightarrow g_{1}\in \left[103,614\right]\;\left(a.u.\right)$$

and

(15) $$y_{d}\left(g_{1}\right)=L_{s}+\frac{\left(3249.7-L_{s}\right)}{1+{\left(g_{1}/150.4\right)}^{2}}\;$$

where *L*_*s*_ denotes the low level of switching or basal level of promoter-RBS component *c*_3_. Note that the standard deviations of parameter fluctuations that are supposed to be tolerated *in vivo* and from environmental disturbances are the same as in (12). Finally, GA is employed to search a set {*c*_3_, *I*_*aTc*_} from corresponding libraries to minimize the following cost function:

(16) $$\begin{array}{l} \min_{\;c_{3}\in \mathrm{Li}b_{\mathrm{Tet}},I_{2}\in \left[2*10^{-3},10\right]}J_{S}\left(c_{3},I_{\mathrm{aTc}}\right)\\ =\min_{\;c_{3}\in \mathrm{Li}b_{\mathrm{Tet}},I_{2}\in \left[2*10^{-3},10\right]}\;E\int_{103}^{614}{\left(y_{\mathrm{ss}}\left(c_{3},I_{\mathrm{aTc}},g_{1}\right)-y_{d}\left(g_{1}\right)\right)}^{2}dg_{1} \end{array}$$

**Figure 3:**
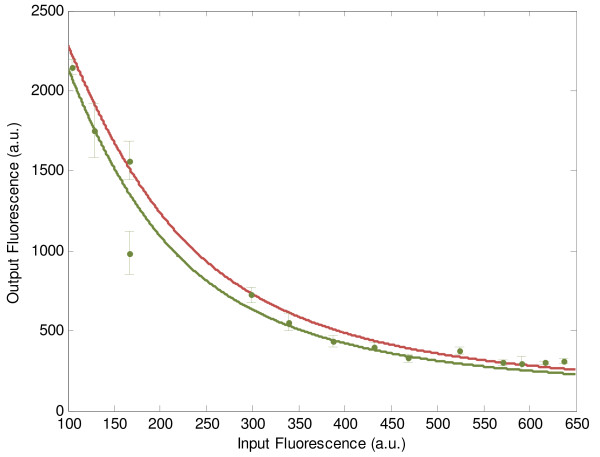
**The switch design example of synthetic genetic transistor.** For switch design example of synthetic genetic transistor, a desired I/O switch response is computed by (15) as shown in red line. The most adequate promoter-RBS component *c*_3_ and aTc concentration *I*_*aTc*_ to fit the desired I/O switch response are searched as {*c*_3_, *I*_*aTc*_} = {*T*_1_, 0 ng/ml} by minimizing *J*_*S*_(*c*_3_, *I*_*aTc*_) in (16) from the corresponding promoter-RBS library *Lib*_*Tet*_ and concentration range of inducer *I*_*aTc*_. The green points are the experimental results, and the error bars are the standard deviations. The green line is the estimated I/O switch response based on experimental data. Obviously, the I/O response of the designed genetic switch could match the desired I/O switch response quite well.

Then, the most adequate promoter-RBS component from the corresponding library and aTc concentrations are found to be {*c*_3_, *I*_*aTc*_} = {*T*_3_, 0 ng/ml}. The estimation of I/O response of genetic transistor based on experimental results is shown in Figure 3, with experimental details summarized in Additional file 1. Clearly, the switching I/O characteristics of synthetic genetic transistor can match the desired I/O response under the intrinsic fluctuations and environmental disturbances.

According to the above examples, the amplification or switching I/O characteristics of a synthetic genetic transistor with different design specifications can be achieved by selecting the most adequate promoter-RBS component *c*_3_ and inducer concentration using the proposed library-based searching method. However, not just the promoter-RBS component *c*_3_ ∊ *Lib*_*Tet*_ can be selected to achieve the amplification or switching design specification of the synthetic genetic transistor circuit, but also other promoter-RBS components, *i.e.*, *Lib*_*Lac*_, can be selected to achieve the desired I/O response. However, for various design specifications, more promoter-RBS libraries are needed to achieve these design specifications.

For the convenience of synthetic genetic transistor design for synthetic biologists, one look-up table has been built for the various design specifications as shown in Table 1 via selecting adequate promoter–RBS components from the corresponding libraries and adequate inducer concentration to achieve the optimal matching in (9). Based on various amplification gains in some specific operation range, the synthetic genetic transistors can be designed by first checking the look-up table. In future, more promoter-RBS components and inducer concentrations for different I/O characteristics of synthetic genetic transistors can be accumulated to build much larger look-up tables to match a lot of design specifications. From this look-up table, based on the desired design specifications, we can select the adequate promoter-RBS components and inducer concentrations to synthesize the genetic transistors with desired I/O responses. Thus, less time will be spent on the design procedure as a designer will be able to easily construct transistors with the desired I/O characteristics.

**Table 1:** The look-up table with different gain specifications for synthetic genetic transistors

**Amplifier gain specifications of synthetic genetic transistor**				
	**Input range (a.u.)**	**Gain**	**Promoter-RBS component**	**Inducer concentration**
*Lib*_*Tet*_	120 ~ 180	-10.00	*T*_3_	0 ng/ml
	150 ~ 225	-7.50	*T*_3_	1 ng/ml
	260 ~ 460	-2.00	*T*_3_	0 ng/ml
	400 ~ 550	-1.00	*T*_3_	1 ng/ml
	460 ~ 560	-0.75	*T*_3_	0 ng/ml
	575 ~ 620	-0.50	*T*_3_	1 ng/ml
*Lib*_*Lac*_	40 ~ 140	-0.15	*L*_1_	0 mM
	40 ~ 140	-2.50	*L*_3_	0 mM

Given the desired amplification gains and their input signal ranges, we could select adequate promoter-RBS component and inducer concentration from the table to achieve the minimum matching error in (9).

## Discussion

One major aim of synthetic biology is to construct a gene circuit with the desired functionality of an organism. Recently, promoter libraries and promoter-RBS libraries have been built to simulate the *in vivo* behavior of a gene circuit [30,38,39]. By identifying the kinetic strengths of promoter-RBS components, the protein expressions in the gene circuit can be estimated and predicted. However, in the process of constructing promoter-RBS library, the identified kinetic parameters in the promoter-RBS library can be affected by several conditions, including the medium, copy number of plasmid, terminator and so on. Therefore, for the extensive application of promoter-RBS libraries, the construction conditions of promoter-RBS libraries need to be unified and standardized. This will allow standardized promoter-RBS libraries, similar to electronic component libraries, which can be easily used and expanded by other gene circuit designers.

In this study, by the promoter-RBS libraries we established, a genetic transistor has been constructed and implemented. Additionally, the synthetic genetic transistor can perform amplification and switching like an electronic transistor according to its I/O characteristics. The I/O characteristics of the synthetic genetic transistor circuit are simulated by a mathematic model with random parameter fluctuation to guarantee the robustness of the design *in vivo*. The design specification of amplification or switching in the genetic transistor can be achieved by the library-searching method using GA. By optimally matching the desired I/O response of amplification or switching, the most adequate set of promoter-RBS component and inducer concentration {*c*_3_, *I*_2_} can be selected to construct a genetic transistor with the desired design specifications. The library-searching method using GA is introduced to reduce the number of trial-and-error attempts, as well as the searching time in libraries when the libraries have a large number of components. Furthermore, for the convenience of synthetic genetic transistor design for synthetic biologists, one look-up table has been built for the various design specifications as shown in Table 1. From this look-up table, based on the desired design specifications, we can select the adequate promoter-RBS components and inducer concentrations to synthesize the genetic transistors with desired I/O responses. Thus, less time will be spent on the design procedure as a designer will be able to easily construct transistors with the desired I/O characteristics.

For applications of the genetic transistor, the various biological components need to be characterized and standardized. By using characterized and standardized genetic components, the design specification of a genetic transistor can be set and the look-up tables can be used to support the genetic circuit design. The genetic transistor described here has a number of potential applications. The amplifier can be used to amplify the oscillation signal reversely and linearly. Based on the designed oscillatory genetic circuits [7,11-13,40-42] in oscillatory metabolic pathways [43-45], an adequate genetic transistor selected from the look-up tables according to the oscillation range and desired amplification gain can be inserted into these circuits directly to amplify the oscillatory signal. In this way, the original genetic circuits do not need to be redesigned. On the other hand, the switch can be used to detect some signals and act like a detector or biosensor [25,29,46,47]. When the input signal changes, the output signal will switch to the other state and make the downstream circuit respond to the signal change. Also, the switch of a genetic transistor can work as logic gates as in an electronic transistor [16-18]. With different combinations of genetic transistors, different logic gates can be constructed.

## Conclusions

In this study, three kinds of libraries, *i.e.*, a constitutive promoter-RBS library, repressor-regulated promoter-RBS library and activator-regulated promoter-RBS library, were established for constructing synthetic gene circuits with the desired transistor amplification or switching function. The amplification gain and switching level of a genetic transistor could be calibrated by selecting adequate promoter-RBS components and inducer concentrations from the corresponding libraries. For the measurement of I/O response, we could embed an additional repressor-regulated promoter-RBS component with reporter protein at the input terminal to measure the input signal while replacing the output protein with a reporter protein to measure the output signal. Further, for the convenience of input regulation, an external circuit was constructed to control the input signal using the concentration of inducer. Based on the desired I/O response in relation to amplification or switching of a genetic transistor, the GA-based searching algorithm was introduced to search for the most appropriate set of promoter-RBS components and inducer concentration from the corresponding promoter-RBS libraries to achieve prescribed I/O characteristics in the genetic transistor. In the simulation results for this study, we demonstrated that the genetic transistor designed here has the prescribed function of amplification or switching. By the library-searching method using GA, different design specifications of amplifier or switch could be achieved the most appropriate set of promoter-RBS components from the corresponding promoter-RBS libraries and inducer concentration within a feasible region. Finally, a look-up table was built for genetic transistor design with different genetic transistor design specifications. Using this table, we could easily select an adequate set of promoter-RBS components and inducer concentration to construct the desired genetic transistor. This innovation saves much time in trial and error attempts in the iterative experimental procedure.

## Competing interests

The authors declare that they have no competing interests.

## Authors’ contributions

BSC formulated the research topic and gave the guideline, YYL and CYH performed the computation and simulation. LJL, CCC, HCC, THY, CL, ZX and RHH performed the experiments. All authors have written and revised the manuscript together. All authors read and approved the final manuscript.

## Supplementary Material

Additional file 1 Supplementary Appendix. Click here for file
