# Supplementary material for: Systematic design methodology for robust genetic transistors based on I/O specifications via promoter-RBS libraries
Source: BMC Syst Biol. 2013 Oct 27;7:109. doi: 10.1186/1752-0509-7-109 (PMC4015965; doi:10.1186/1752-0509-7-109)
Supplement: Additional file 1 — Supplementary Appendix. [file 1752-0509-7-109-S1.pdf]

Appendix

Appendix A: Construction of promoter-RBS libraries

Construction of constitutive promoter-RBS libraries

Before we construct the promoter-RBS libraries, we select the GFP (BBa_E0040) to measure the fluorescence of protein. The fluorescence of GFP can respond to the upstream signal variations. For the construction of the constitutive promoter-RBS libraries, we choose 3 constitutive promoters, *i.e.*, BBa_J23101, BBa_J23105, BBa_J23106, and 3 RBSs, *i.e.*, BBa_B0031, BBa_B0032, BBa_B0034, to construct constitutive promoter-RBS components.

After a constitutive promoter-RBS component is inserted at the upstream of the reporter protein as shown in Figure A1(i), the reporter protein could be produced continually. The reporter protein could be diluted by cell growth, degraded by protease and matured by the post-translation modification. Similarly, the mature reporter protein can be diluted and degraded, too. The dynamic model of reporter protein under the regulation of constitutive promoter-RBS component with the environmental disturbances is shown as follows [32, A1].

$$\begin{aligned} \dot{x}(c,t) &= p_{const}(P_c) - (m+\mu+\gamma_{im,x})x(c,t) + v_1(t) \\ \dot{g}(c,t) &= mx(c,t) - (\mu+\gamma_{m,x})g(c,t) + v_2(t) \end{aligned} \quad \text{(A1)}$$

where $x(c,t)$ and $g(c,t)$ denote the concentrations of the immature and mature reporter protein, respectively; $v_1(t)$ and $v_2(t)$ denote the environmental disturbances of the immature and mature reporter protein, respectively; μ denotes the dilution rate, m denotes the mature rate of the reporter protein, and $\gamma_{im,x}$ and $\gamma_{m,x}$ denote the degradation rates of the immature and mature reporter protein , respectively.

The procedure for constructing constitutive promoter-RBS libraries shown in Figure A1 can be divided into four steps: (i) select the candidate constitutive

promoter-RBS components and insert into the upstream of the reporter gene, (ii) measure the time-profile of the fluorescence of the reporter protein and the cell density, (iii) identify the dilution rate μ and the kinetic strength P_c of the promoter-RBS component by (A1), and (iv) collect P_c for the indexes of the constitutive promoter-RBS library. All of the constitutive promoter-RBS components are listed in Table A1. And the constitutive promoter-RBS library is shown in Table A4.

Construction of repressor and activator-regulated promoter-RBS libraries

The construction of regulated promoter-RBS libraries is more complex than the construction of constitutive promoter-RBS libraries. The regulated promoter-RBS components have different kinetic strengths with or without saturated concentrations of the inducer. Hence, the relationship between the concentrations of inducer and the regulated promoter-RBS component is considered for the characterization of the regulated promoter-RBS components. In general, the regulated promoters can be divided into two major types, *i.e.*, the repressor-regulated and activator-regulated promoter, as shown in Figures A2(iv) and A3(i), respectively. In Figures A2(iv) and A3(i), a constitutive promoter-RBS component is used to produce the corresponding regulator continually for the regulator-regulated promoter-RBS component, and the corresponding inducer would be added to bind the corresponding regulator, and then regulate the promoter-RBS component. For the repressor-regulated promoter-RBS components, when the corresponding repressor is produced by the constitutive promoter-RBS component, the repressor-regulated promoter-RBS component will be repressed and the production of protein at the downstream of repressor-regulated promoter-RBS component will be decreased, too. However, when the corresponding inducer is added, the inducer will bind the repressor and restrain the repressor from

binding the promoter-RBS component. As the promoter-RBS component will not be repressed again, the production of protein will increase. In this situation, the full dynamic model of repressor-regulated promoter-RBS component in Figure A2(iv) can be described as follows

$$\begin{aligned}
\dot{x}_{repressor}(c_i,t) &= p_{const}\left(P_{c_i}\right)-\left(\mu+\gamma_{repressor}\right)x_{repressor}(c_i,t)\\
\dot{x}\left(c_j,t\right) &= p_{repressor}\left(P_{M,c_j},P_{m,c_j},x_{repressor},I_{inducer}\right)-\left(m+\mu+\gamma_{im,x}\right)x\left(c_j,t\right)\\
\dot{g}\left(c_j,t\right) &= mx\left(c_j,t\right)-\left(\mu+\gamma_{m,x}\right)g\left(c_j,t\right)
\end{aligned} \tag{A2}$$

where c_i and c_j denote the components of the constitutive and repressor-regulated promoter-RBS libraries, respectively; $\gamma_{repressor}$ denotes the degradation rate of the repressor. The regulatory function can be described as follows:

$$p_{repressor}\left(P_{M,c_2},P_{m,c_2},x_{repressor},I_1\right)=P_{m,c_2}+\frac{P_{M,c_2}-P_{m,c_2}}{1+\left(x^*_{repressor}/K_{repressor}\right)^{n_{repressor}}}$$

with $x^*_{repressor}=\dfrac{x_{repressor}}{1+\left(I_1/K_{inducer}\right)}$

where P_{M,c_2} and P_{m,c_2} denote the maximum and minimum kinetic strengths of repressor-regulated promoter-RBS component c_2, respectively, $n_{repressor}$ denotes the binding cooperativity between repressor and DNA, $K_{repressor}$ and $K_{inducer}$ denote the repressor-DNA binding affinity and inducer-repressor dissociation rate, respectively, and $x^*_{repressor}$ denotes the repressor activity.

On the other hand, for the activator-regulated promoter-RBS components, the promoter-RBS strength can maintain a very low or no basal level and finally protein is not produced as the corresponding activator and inducer do not exist. However, when the activator is produced and the inducer is added, the inducer will bind the activator to form a complex and activate the promoter-RBS component to increase its kinetic strength. Then, the full dynamic model of activator-regulated promoter-RBS component in Figure A3(i) can be described as follows

$$
\begin{aligned}
&\dot{x}_{activator}(c_i,t) = p_{const}\left(P_{c_i}\right) - \left(\mu + \gamma_{activator}\right) x_{activator}(c_i,t) \\
&\dot{x}(c_k,t) = p_{activator}\left(P_{M,c_k}, P_{m,c_k}, x_{activator}, I_{inducer}\right) - \left(m + \mu + \gamma_{im,x}\right) x(c_k,t) \\
&\dot{g}(c_k,t) = m x(c_k,t) - \left(\mu + \gamma_{m,x}\right) g(c_k,t)
\end{aligned}
\tag{A3}
$$

where c_i and c_k denote the components of the constitutive and activator-regulated promoter-RBS libraries, respectively; $\gamma_{activator}$ denotes the degradation rate of the activator. The regulatory function can be described as follows:

$$
p_{activator}\left(P_{M,c_3}, P_{m,c_3}, x_{activator}, I_2\right) = P_{m,c_3} + \frac{\left(P_{M,c_3} - P_{m,c_3}\right)\cdot\left(x^*_{activator}\right)^{n_{activator}}}{\left(x^*_{activator}\right)^{n_{activator}} + \left(K_{activator}\right)^{n_{activator}}}
$$

$$
\text{with } x^*_{activator} = \frac{x_{activator}\cdot I_2}{I_2 + K_{inducer}}
$$

where P_{M,c_3} and P_{m,c_3} denote the maximum and minimum kinetic strengths of activator-regulated promoter-RBS component c_3, respectively, $n_{activator}$ denotes the binding cooperativity between activator and DNA, $K_{activator}$ denotes the activator-DNA binding affinity, and $x^*_{activator}$ denotes the activator activity.

Since different concentrations of inducer will produce different fluorescences of the reporter protein, the relationship between the concentration of inducer and the kinetic strength needs to be considered for the construction of regulated promoter-RBS libraries. Not only the time profile data of the fluorescence but also the relationship between the concentration of inducer and the fluorescence are needed for identifying the kinetic strengths of regulated promoter-RBS components more completely.

The procedure for the construction of repressor-regulated promoter-RBS libraries can be summarized in Figure A2. First, we need to select the candidate repressor-regulated promoter-RBS components to be inserted into the upstream of the reporter protein. Then we measure the time-profile data of the fluorescence and identify the maximum promoter-RBS strength P_{M,c_j} of c_j, like the procedure of constructing constitutive promoter-RBS library. Finally, we need to select a suitable

constitutive promoter-RBS component to produce the repressor continually in the gene circuit as shown in Figure A2(iv). Since different concentrations of inducer will produce different concentrations of fluorescence of the reporter protein at the steady state, the fluorescences with different concentrations of inducer at the steady state need to be measured. With these data and P_{M,c_j}, we can use the steady state model to identify other parameters, *i.e.*, P_{m,c_j}, $n_{repressor}$, $K_{repressor}$ and $K_{inducer}$. Finally, all of the identified parameters are collected for the indexes of the repressor-regulator promoter-RBS libraries.

On the other hand, the construction procedure of the activator-regulated promoter-RBS libraries is different from the repressor-regulated promoter-RBS libraries. For the measurement of activator-regulated promoter-RBS components, it should be noted that when no inducer or activator exists, there is no fluorescence production or low basal fluorescence which couldn't be measured by the instrument. Hence, the measurement method needs to be modified for the characterization of the activator-regulated promoter-RBS components. The construction procedure of the activator-regulated promoter-RBS libraries is shown in Figure A3. First, we select the candidate activator-regulated promoter-RBS components and construct the gene circuit as shown in Figure A3(i). Then, we choose some specific concentrations of inducer to measure the time-profile data of fluorescence. In order to more accurately characterize the activator-regulated promoter-RBS components, different concentrations of inducer are added to produce different fluorescences at the steady state to measure different time-profile data of fluorescence. Finally, based on different fluorescences at steady state and inducer concentrations, we can use the steady state model to identify the kinetic parameters, *i.e.*, P_{M,c_k}, P_{m,c_k}, $n_{activator}$, $K_{activator}$ and $K_{inducer}$. And all of the identified kinetic parameters are collected for the indexes of the

activator-regulator promoter-RBS libraries.

The repressor-regulated promoter-RBS libraries and activator-regulated promoter-RBS libraries are shown in Table A5 and Table A6, respectively. In the repressor-regulated promoter-RBS libraries, we select 2 repressor-regulated promoters, *i.e.*, aTc-responsive promoter P_{tetR}, and IPTG-responsive promoter P_{lacI} and 3 RBSs which are the same as the previous promoter-RBS libraries; In the activator-regulated promoter-RBS libraries, we select 1 activator-regulated promoters, *i.e.*, 3oC6HSL-responsive promoter P_{lux} and 2 RBSs which are the same as the previous promoter-RBS libraries. All of the regulated promoter-RBS components and the corresponding regulators are listed in Table A2.

References

A1. Leveau JHJ, Lindow SE: **Predictive and Interpretive Simulation of Green Fluorescent Protein Expression in Reporter Bacteria.** *J Bacteriol.* 2001, **183:**6752-6762.

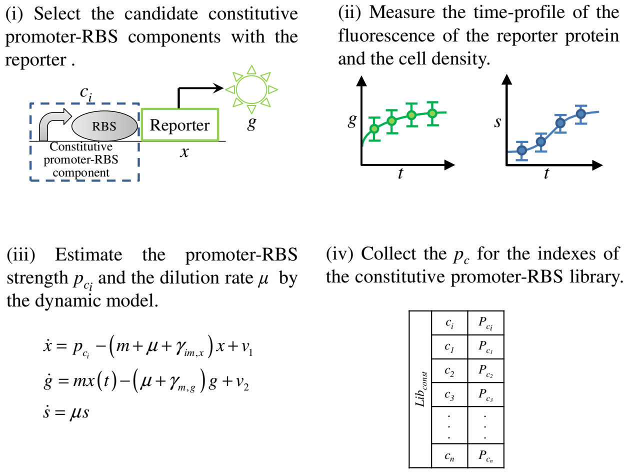

Figure A1 - The construction procedure (i)-(iv) for constitutive promoter-RBS library Lib_{const}: (i) The constitutive promoter-RBS component c_i is constructed at the upstream of reporter gene. (ii) The time-profiles of fluorescence of reporter protein and cell density are measured. (iii) The dynamic model is used to identify the kinetic strength P_{c_i} and dilution rate μ. (iv) The constitutive promoter-RBS library Lib_{const} is constructed based on the kinetic strengths of constitutive promoter-RBS components.

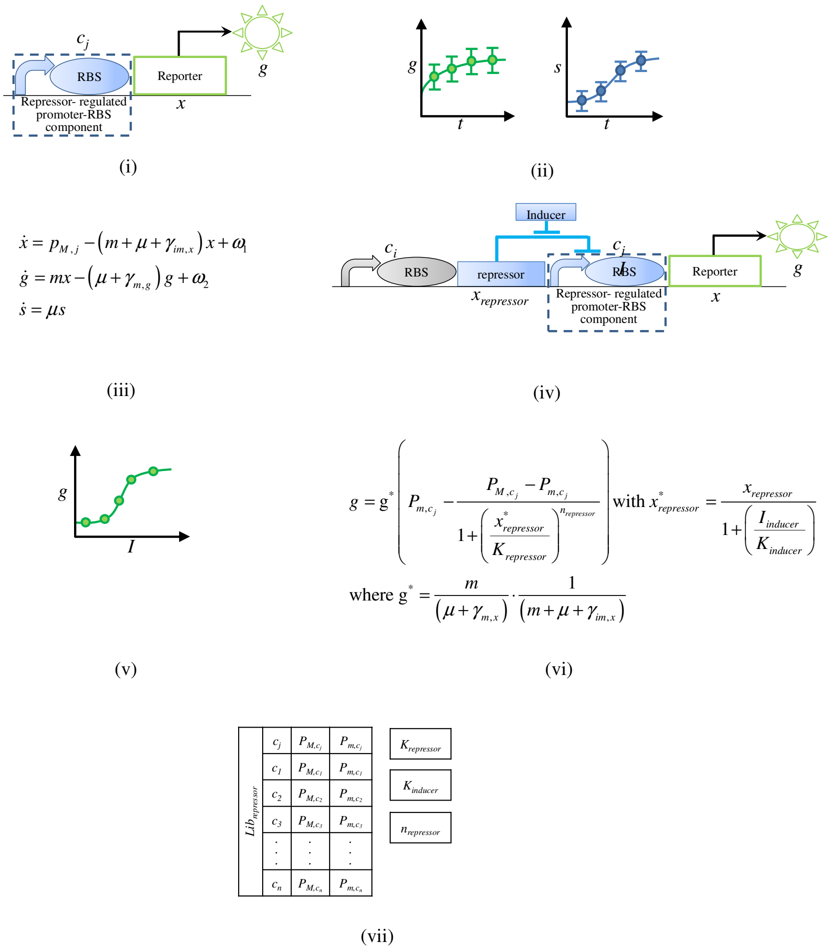

Figure A2 - The construction procedure (i)-(vii) for repressor-regulated promoter-RBS library $Lib_{repressor}$: (i) The repressor-regulated promoter-RBS component c_j is constructed at the upstream of reporter gene. (ii) The time-profiles of fluorescence of reporter protein and cell density are measured. (iii) The dynamic model is used to identify the kinetic strength P_{M,c_j} and dilution rate μ. (iv) The repressor $x_{repressor}$ with constitutive promoter-RBS component c_i is constructed to repress the repressor-regulated promoter-RBS component c_j. (v) Different concentrations of inducer I are added to measure the fluorescence at steady state. (vi) The Hill function is used to identify the parameters at steady state, *i.e.*, P_{m,c_j}, $n_{repressor}$, $K_{repressor}$ and $K_{inducer}$. (vii) The repressor-regulated promoter-RBS library $Lib_{repressor}$ is constructed based on the kinetic strengths of repressor-regulated promoter-RBS components.

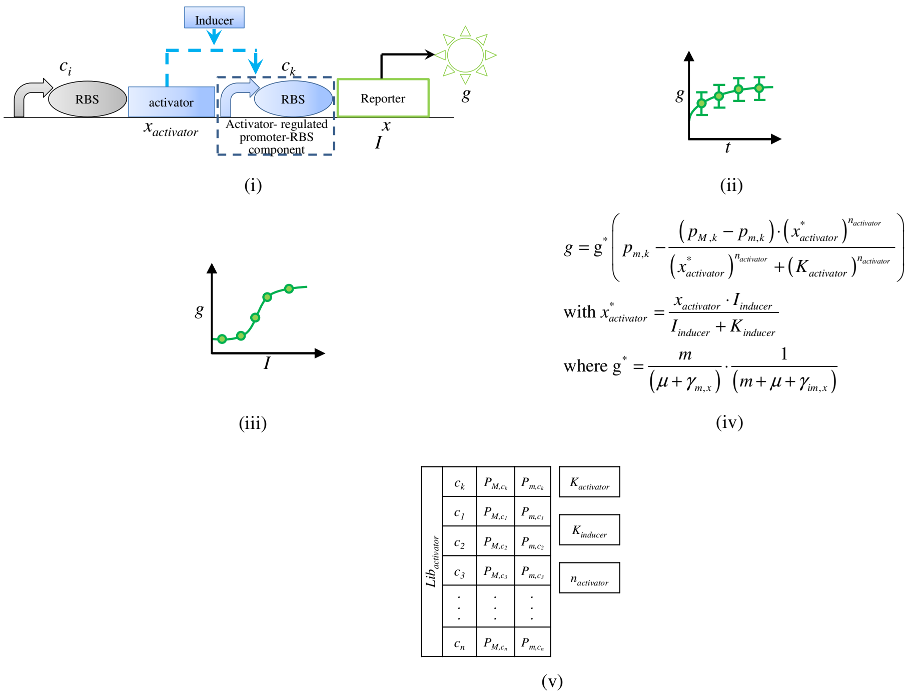

Figure A3 - The construction procedure of activator-regulated promoter-RBS library $Lib_{activator}$: (i) The activator-regulated promoter-RBS component c_k is constructed at the upstream of reporter gene. And the activator $x_{activator}$ with constitutive promoter-RBS component c_i is constructed to activate the activator-regulated promoter-RBS component c_k when the inducer I is added. (ii) The least two different concentrations of inducer are provided to measure the time-profiles of fluorescence of reporter. (iii) Different concentrations of inducer I are added to measure the fluorescence at steady state. (iv) The Hill function is used to identify the parameters at steady state, *i.e.*, P_{M,c_k} , P_{m,c_k} , $n_{repressor}$, $K_{repressor}$ and $K_{inducer}$. (v) The activator-regulated promoter-RBS library $Lib_{activator}$ is constructed based on the kinetic strengths of activator-regulated promoter-RBS components.

Table A1 List of the candidate constitutive promoters and RBSs in the BioBricks. The constitutive promoters will combine with the RBSs as the constitutive promoter-RBS components in this study. All BioBrick parts refer to Registry of Standard Biological Parts (http://partsregistry.org/Main_Page)

**Property**	**Biobrick No.**	**Description**
Constitutive promoter	BBa_J23100	Parts J23100 through J23119 are a family of constitutive promoter parts isolated from a small combinatorial library. In this study, we only take five parts to be characterized, *i.e.*, BBa_J23101, BBa_J23105, BBa_J23106.
	BBa_J23101	
	BBa_J23102	
	BBa_J23103	
	BBa_J23104	
	BBa_J23105	
	BBa_J23106	
	BBa_J23107	
	BBa_J23108	
	BBa_J23109	
	BBa_J23110	
	BBa_J23111	
	BBa_J23112	
	BBa_J23113	
	BBa_J23114	
	BBa_J23115	
	BBa_J23116	
	BBa_J23117	
	BBa_J23118	
Ribosome binding site (RBS)	BBa_B0031	BBa_B0031, BBa_B0032 and BBa_B0034 are all ribosome binding sites with different strengths.
	BBa_B0032	
	BBa_B0034	

Table A2 List of the candidate regulated promoters, repressors and activators in the BioBrick. All BioBrick parts refer to Registry of Standard Biological Parts (http://partsregistry.org/Main_Page)

BioBrick No.		Repressor/ Activator	Inducer	Description
Repressor-regulated promoter	BBa_R0040 (P_{Tet})	BBa_C0040 (TetR)	Anhydrotetracycline (ATc)	The promoter is constitutively ON and repressed by the repressor TetR.
	BBa_R0010 (P_{Lac})	BBa_C0010 (LacI)	Isopropyl-beta-D-thiogalactopyranoside (IPTG)<br>Lactose	The promoter contains two protein binding sites. One is the CAP protein binding site, and the other is LacI protein binding site. The promoter is repressed when the CAP or LacI protein binds to each binding site, respectively.
Activator-regulated promoter	BBa_R0062 (P_{Lux})	BBa_C0062 (LuxR)	3oC6HSL	The promoter has a weak constitutive expression of downstream genes. This expression is up-regulated by the action of the LuxR activator protein complexed with the autoinducer 3oC6HSL.

Table A3 List of reporter protein, terminator and backbone. All BioBrick parts refer to Registry of Standard Biological Parts (http://partsregistry.org/Main_Page)

**Property**	**BioBrick No.**	**Description**
Reporter protein	BBa_E0040	Protein GFPmut3b. Green fluorescent protein derived from jellyfish *Aequeora Victoria* wild-type GFP.
	K145015	Protein GFPmut3b+LVA. Green Fluorescent Protein with LVA tag for rapid degradation.
Backbone	pSB3K3	Low to medium copy numbers of BioBrick standard vector
Terminator	BBa_B0015	Double terminator consisting of BBa_B0010 and BBa_B0012. This is the most commonly used terminator.

Table A4 The constitutive promoter-RBS library Lib_{const}. The kinetic strengths of constitutive promoter-RBS components are identified with 95% confidence from experiment data as the indexes of constitutive promoter-RBS library Lib_{const}.

Symbol	Plasmid	Promoter-RBS kinetic strength
J_1	J23101-B0031	94.520
J_2	J23101-B0032	65.892
J_3	J23101-B0034	237.000
J_4	J23105-B0031	14.493
J_5	J23105-B0032	6.512
J_6	J23105-B0034	28.586
J_7	J23106-B0031	22.011
J_8	J23106-B0032	12.193
J_9	J23106-B0034	58.781

Table A5 The repressor-regulated promoter-RBS library $Lib_{repressor}$**.** The kinetic strengths of repressor-regulated promoter-RBS components are identified with 95% confidence from experiment data as the indexes of repressor-regulated promoter-RBS components $Lib_{repressor}$.

Symbol		BioBrick part	p_M	p_m	Other kinetic parameters
Lib_{Tet}	T_1	R0040-B0031	11.965	3.031	K_{atc} = 6.794 ng/ml<br>K_{TetR} = 45.130 nM<br>n_{TetR} = 2
	T_2	R0040-B0032	8.740	3.381	
	T_3	R0040-B0034	162.557	4.837	
Lib_{Lac}	L_1	R0010-B0031	3.859	2.039	K_{IPTG} = 0.008 mM<br>K_{LacI} = 27.484 nM<br>n_{LacI} = 2
	L_2	R0010-B0032	2.586	2.529	
	L_3	R0010-B0034	33.934	1.495	

Table A6 The activator-regulated promoter-RBS library $Lib_{activator}$**.** The kinetic strengths of repressor-regulated promoter-RBS components are identified with 95% confidence from experiment data as the indexes of repressor-regulated promoter-RBS components $Lib_{activator}$.

Symbol		BioBrick part	p_M	p_m	Other kinetic parameters
Lib_{Lux}	U_1	R0062-B0031	29894.75	19.927	$K_{3OC6HSL}$ = 3.345 nM<br>K_{LuxR} = 0.307nM<br>n_{LuxR} = 1
	U_2	R0062-B0032	20388.03	34.073	

Table A7 The kinetic parameters of transistor dynamic model.

Kinetic parameter	Description	Value	Unit	Ref.
γ_m	GFP degradation rate	0.35007×10^{-3}	min^{-1}	[48]
γ_{im}	Immature GFP degradation rate	5.7762×10^{-3}	min^{-1}	[49]
m	Maturation rate	5.7762×10^{-3}	min^{-1}	[49]
γ_{TetR}	TetR degradation rate	0.1386	min^{-1}	[42]
γ_{LacI}	LacI degradation rate	0.1386	min^{-1}	[42]
μ	Dilution rate due to cell growth	0.011946	min^{-1}	*

* The values are identified from the experiments.

Appendix B: The operation of an electronic transistor

A transistor is a semiconductor device used for different functions in electronic circuits, such as signal amplification and switching. It has the advantages of low volume, high efficiency, a long life-span and high speed switching. Two major types of transistor are the bipolar junction transistor (BJT) and metal-oxide-semiconductor field-effect transistor (MOSFET). The functions of these two types of transistors are similar. The electronic signal can be amplified linearly or switched by controlling the bias voltage. Considering the BJT for example, a three-terminal npn BJT has three terminal connections which are called the emitter, base and collector, as shown in Figure B1(a) [1]. The complete npn BJT circuit in the common-emitter configuration is shown in Figure B2(b), in which the electronic signal from base terminal i_B can be amplified linearly or switched through BJT by controlling the voltage between the collector and emitter terminals V_{CE} in the linear region larger than 0.3V or saturation region smaller than 0.3V. When V_{CE} is controlled in the linear region, i_B is amplified linearly. The signal output from collector terminal i_C is almost equal to $\beta \cdot i_B$ and the variation in input voltage V_{in} is also amplified reversely and linearly, where the parameter β is the common-emitter current gain within the range of 50 to 300. On the other hand, when V_{CE} is controlled in the saturation region, the output signal will be switched such that a high level signal will be switched into a low level signal and vice versa [B1-B2]. The mathematical models to describe the behaviors of an npn BJT circuit in the common-emitter configuration are shown as follows.

In the cut-off region: $V_{in} \le V_{BE(on)}$

$$V_o = V_{CC} \tag{B1}$$

In the forward-active region (linear region): $V_{in} > V_{BE(on)}$

$$V_o = -\beta \frac{R_2}{R_1}\left(V_{in} - V_{BE(on)}\right) + V_{CC} \tag{B2}$$

In the saturation region: $V_{in} \geq \frac{R_1}{\beta \cdot R_2}\left(V_{CC} - V_{CE(sat)}\right) + V_{BE(on)}$

$$V_o = V_{CE(sat)} \qquad \text{(B3)}$$

where $V_{BE(on)}$ and V_{CC} denote the turn-on and bias voltages of the npn BJT circuit, respectively, and $V_{CE(sat)}$ denotes the voltage between collector and emitter in saturation; R_1 and R_2 are the values of the resistance. The current-voltage and voltage I/O characteristics of the npn BJT circuit in the common-emitter configuration are shown in Figures B2(a) and B3(b), respectively, and the voltage I/O characteristics of electronic transistor is shown in Figure B3.

References

B1. Neamen DA: *Microelectronics: Circuit Analysis and Design*. McGraw-Hill; 2007.

B2. Varadarajan PA, Del Vecchio D: **Design and Characterization of a Three-terminal Transcriptional Device Through Polymerase Per Second.** *IEEE Trans NanoBioscience.* 2009, **8:**281-289.

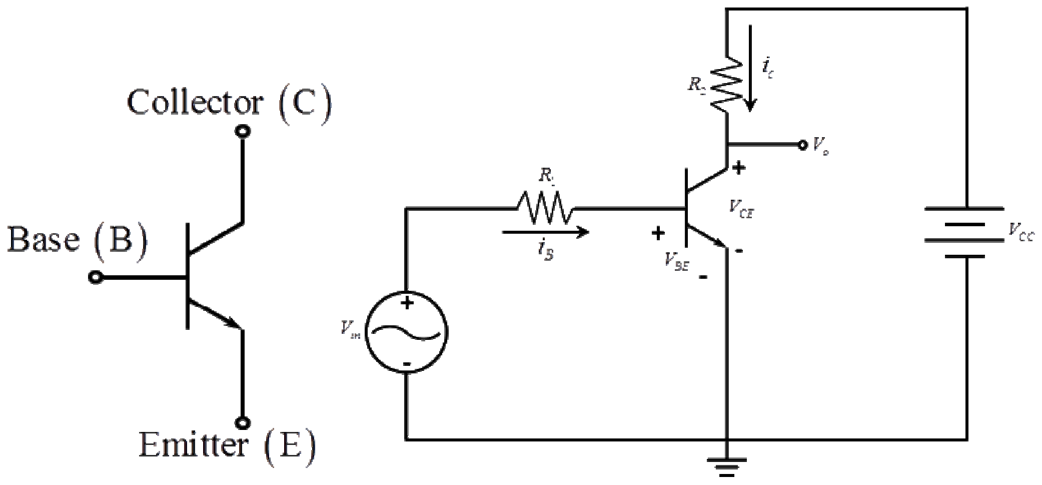

(a) (b)

Figure B1 - The npn bipolar junction transistor: (a) The npn BJT circuit symbol. BJT contains three terminal connections, *i.e.*, the so- called Base, Collector and Emitter. (b) The npn BJT circuit in the common-emitter configuration.

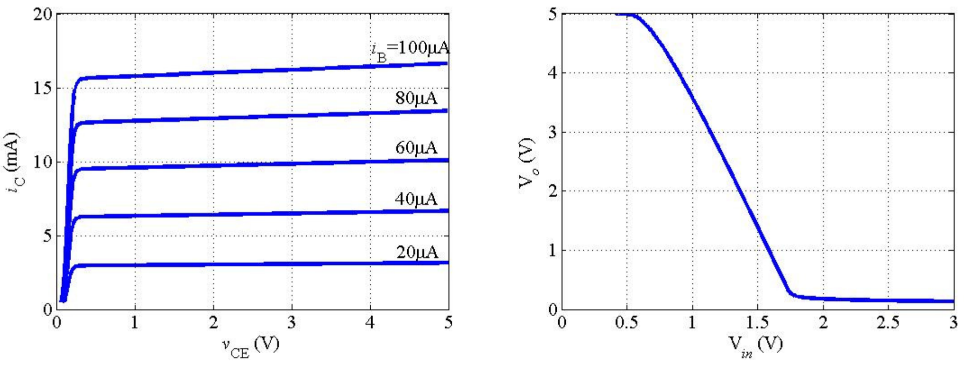

(a) (b)

Figure B2 - The current-voltage characteristic and voltage I/O characteristic of BJT in the common-emitter configuration: The BJT circuit is shown in Figure B1(b), and its characteristics are simulated by the PSpice with a standard 2N3904 transistor from PSpice library. (a) The current-voltage characteristic: The base current i_B changes from 20 μA to 100 μA . (b) The voltage I/O characteristic: Set R_1 =5kΩ , R_2 =150kΩ , V_{CC} =5V, $V_{BE(on)}$ =0.7V, $V_{CE(sat)}$ =0.2V and the input voltage V_{in} from 0 V to 3 V. For $V_{in} \le 0.7$V, the transistor is cut off as shown in (B1); for 0.7 V $< V_{in} <$ 1.9V , the transistor is in the region of the linear amplification as shown in (B2); and for $V_{in} \ge 1.9$V, the transistor is in the saturation region as shown in (B3) [B1].

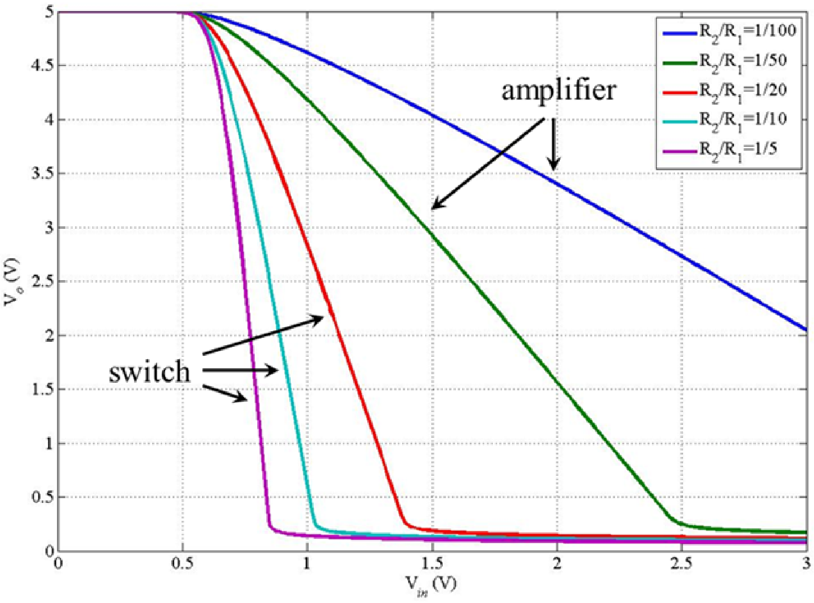

Figure B3 - The voltage I/O characteristics of the common-emitter circuit for different R_2/R_1 ratio: The circuit is shown in Figure B1(b), and simulated by the PSpice with a standard 2N3904 transistor from PSpice library. The voltage I/O characteristics are simulated by changing the R_2/R_1 ratio. According to (B2), when the R_2/R_1 ratio becomes large, the voltage I/O characteristic is much sharper as the amplifier in linear region. And when the R_2/R_1 ratio is large enough, the voltage I/O characteristic will be like a switch.

Appendix C: Materials and methods

Reagents

All restriction enzymes and DNA ligation kit were purchased from New England Biolabs. The chemicals used in this study were ordered from Sigma-Aldrich. Oligonucleotides were ordered from Integrated DNA Technologies.

Bacteria strains and medium

E. coli DH5α cells from Yeastern biotech (ECOS) were used for the constructing procedure. E. coli MG1655 ΔrecA cells were used for the fluorescence measurable experiments. M9 working medium (34 g/L Na2HPO4, 15 g/L K2HPO4, 2.5 g/L NaCl, 5g/L NH4Cl, 10 g/L casamino acids, 2 μM vitamin B1, 2 mM MgSO4, 0.2% Glucose) with proper antibiotics was used for E. coli cultivation at 37°C at 200 r.p.m..

Plasmid construction

The plasmids used in this study were from Biobricks and constructed by the standard iGEM assembly and the DNA devices, including promoters, RBS, report gene, and terminator, were listed in Table 1. All the DNA parts were constructed into the backbone pSB3K3 (20~30 copies per cell).

Fluorescence measurement

For fluorescence determination, cell containing the genetic circuit were inoculated in M9 working medium with 50 mg/mL Kanamycin. After 14~16 hours incubation, the culture was diluted 500-fold and further incubated until the OD600 reached 0.1. Then different concentrations of IPTG (Isopropyl β-D-1-thiogalactopyranoside) and aTc (Anhydrotetracycline) were added into the medium. And the fluorescence intensity of the culture was subsequently measured by the microplate reader (BioTek, Synergy™ H1, GFP settings were 490/530 nm for excitation and emission).

Normalization

For normalizing RFP expression level, a normalization factor is calculated by

$$NF = \frac{G_{ss}}{R_{ss}} \quad \text{(C1)}$$

where G_{ss} and R_{ss} are the steady-state intensity levels of *gfp* and *rfp* for a promoter (e.g., BBa_J23101), respectively. Then the normalized RFP expression level can be given by $g_1 = NF * r$, in which r is the original RFP expression level.
